# Correction: Karayannis et al. Screening of New Industrially Important Bacterial Strains for 1,3-Propanediol, 2,3-Butanediol and Ethanol Production through Biodiesel-Derived Glycerol Fermentations. *Microorganisms* 2023, *11*, 1424

**DOI:** 10.3390/microorganisms13020407

**Published:** 2025-02-13

**Authors:** Dimitris Karayannis, Gabriel Vasilakis, Ioannis Charisteidis, Alexandros Litinas, Eugenia Manolopoulou, Effie Tsakalidou, Seraphim Papanikolaou

**Affiliations:** 1 Department of Food Science and Human Nutrition, Agricultural University of Athens, 75 Iera Odos, 11855 Athens, Greece; dimika96@icloud.com (D.K.); vasilakis@aua.gr (G.V.); mae@aua.gr (E.M.); et@aua.gr (E.T.); 2 Verd S.A., 2nd Industrial Area of Volos, 37500 Velestino, Greece; gcharisteidis@verd.gr (I.C.); alitinas@verd.gr (A.L.)

In the original publication [1], there was a mistake in Figure 1 as published. There was a tiny but very important mistake. An arrow was not pointing in the right direction, meaning the information given for the metabolic pathway was not accurate. The corrected Figure 1 appears below. The authors state that the scientific conclusions are unaffected. This correction was approved by the Academic Editor. The original publication has also been updated.

## Figures and Tables

**Figure 1:**
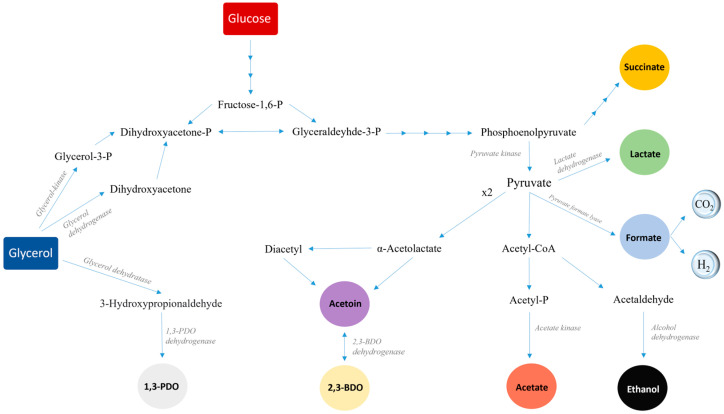
Metabolic pathways of glycerol fermentation by bacterial strains of the genera *Klebsiella*, *Citrobacter*, *Enterobacter* and *Hafnia*.
